# Single-cell atlas of multilineage cardiac organoids derived from human induced pluripotent stem cells

**DOI:** 10.1093/lifemedi/lnac002

**Published:** 2022-06-14

**Authors:** Fengzhi Zhang, Hui Qiu, Xiaohui Dong, Xiaoyan Zhang, Chunlan Wang, Xin Li, Xingwu Zhang, Jie Na, Jin Zhou, Changyong Wang

**Affiliations:** Beijing Institute of Basic Medical Sciences, Beijing 100850, China; School of Life Sciences, Tsinghua University, Beijing 100084, China; Beijing Institute of Basic Medical Sciences, Beijing 100850, China; Department of Ultrasound, Chinese Academy of Medical Sciences and Peking Union Medical College Hospital, Beijing 100084, China; Beijing Institute of Basic Medical Sciences, Beijing 100850, China; Core Laboratory of Translational Medicine, Chinese PLA General Hospital, Beijing 100730, China; School of Medicine, Tsinghua University, Beijing 100084, China; School of Medicine, Tsinghua University, Beijing 100084, China; Beijing Institute of Basic Medical Sciences, Beijing 100850, China; Beijing Institute of Basic Medical Sciences, Beijing 100850, China

**Keywords:** human induced pluripotent stem cells, cardiomyocytes, mini-cardiac organoid, single-cell analysis, myocardial infarction

## Abstract

Human induced pluripotent stem cell (hiPSC)-derived cardiac organoids can be used to model human heart development and cardiovascular disease, and provide therapeutic cells to repair the heart. We used single-cell transcriptome analysis to dissect the development of 3D mini-cardiac organoids (MCOs) consisting of hiPSC-derived cardiomyocytes, and endothelial and smooth muscle cells. We found that the 3D matrix-rich microenvironment significantly promoted the maturation of cardiomyocytes, and mixing endothelial and smooth muscle cells with cardiomyocytes led to the formation of cardiac fibroblast highly expressing *DLK1*. Modulation of DLK1 signaling affected immunomodulatory gene expression in 2D cultured cardiomyocytes. Transplantation of multilineage MCO into a rat model of myocardial infarction significantly improved cardiac function and reduced fibrosis in the infarcted area. Our single-cell analysis of MCO provided rich information about cell state and fate dynamics in the 3D multilineage microenvironment and brought new insight into the molecular mechanism that promotes cardiomyocyte maturation and heart repair.

## Introduction

Irreversible loss and pathological changes in cardiomyocytes (CMs) and fibroblasts after myocardial infarction (MI) eventually lead to heart failure [1, 2]. Improving the hostile tissue microenvironment and replenishing the lost functional cells are critical to repair the failing hearts. Human induced pluripotent stem cells (hiPSCs) can produce large numbers of CMs (hPSC-CMs), endothelial cells (hPSC-ECs), and smooth muscle cells (hPSC-SMCs), owing to their remarkable self-renewal and differentiation potential. These hiPSC-derived cardiovascular cells have been transplanted to infarcted hearts of animal models and showed tremendous regenerative potential. However, their immaturity and heterogeneity posed hurdles for their application in clinical medicine. A wide range of tissue engineering approaches has been employed to improve the physiological function, survival ability, and repair potentials of *in vitro* differentiated CMs. Engineered tissue patches enhanced the structural and functional maturation of hiPSC-derived CMs [3–5]. Upon co-culture with ECs and SMCs, CMs became more resilient to a hypoxic environment [6, 7]. However, how the cell property and behavior change in a complex 3D microenvironment consisting of extracellular matrix (ECM) and different cardiovascular cell types is unclear.

Here, we used single-cell RNA-seq (scRNA-seq) to address this important question. Interestingly, a large proportion of input ECs and SMCs became fibroblasts in the mini-cardiac organoid (MCO) and highly expressed *DLK1*, a non-canonical NOTCH ligand. Finally, we demonstrated that the transplantation of MCO generated by mixing hiPSC-derived CMs, ECs, and SMCs had a strong regeneration effect and improved cardiac function in a rat model of MI.

## Results

### Single-cell analysis of CMs differentiated in 2D

**Figure 1.**
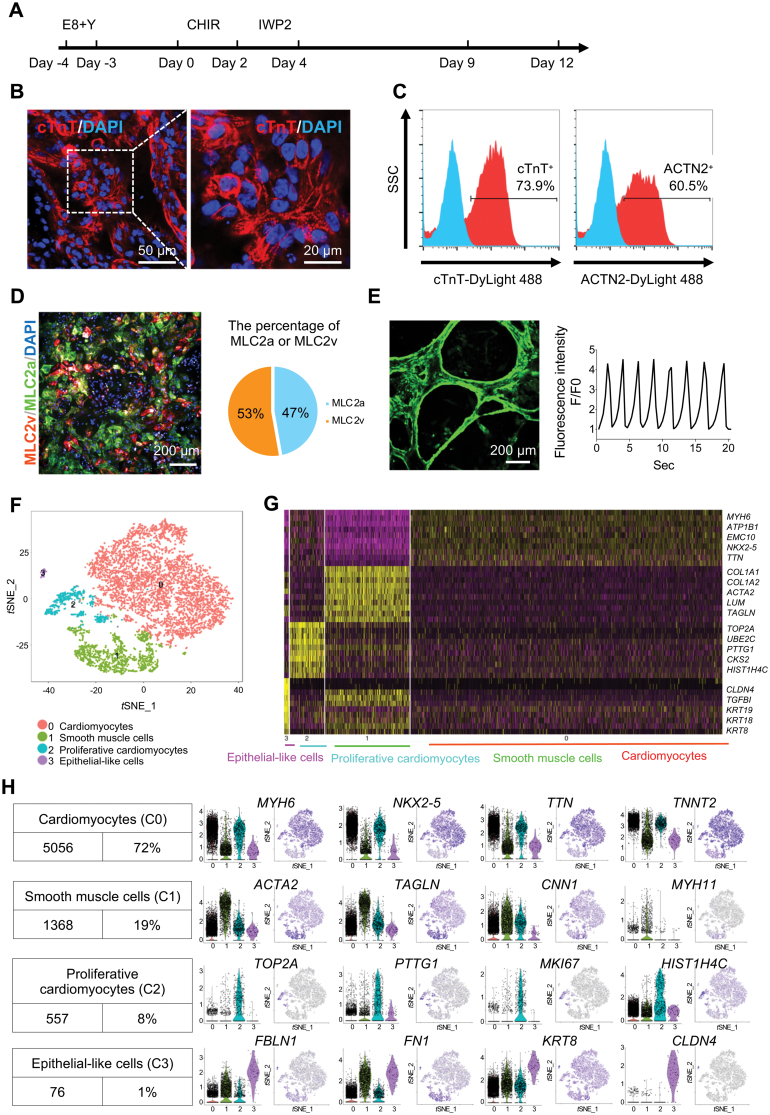
ScRNA-seq analysis of hiPSC-derived CMs from 2D differentiation. (A) A schematic diagram showing the protocol for monolayer-based differentiation from hiPSCs to hiPSC-CMs (2D_CMs). (B) Representative immunofluorescent images showing day 21 2D_CMs positive for cTnT. Scale bars, 50 μm (left) and 20 μm (right). (C) Representative flow cytometric histogram showing protein levels of cTnT and α-actinin (ACTN2) in day 21 2D_CMs. (D) Representative immunofluorescent images of MLC2a and MLC2v in day 21 2D_CMs (left), and then the percentage of cells positive for MLC2a and MLC2v was calculated, respectively (right). Scale bar, 200 μm. (E) Simultaneous confocal imaging of Ca^2+^ transient in day 21 2D_CMs. The left picture corresponds to a single frame of Video S1, showing Ca^2+^ flux in spontaneously beating 2D_CMs. Scale bar, 200 μm. (F–H) ScRNA-seq analysis identifying cell types in 2D_CM. (F) *t*-distributed stochastic neighbor embedding (*t*-SNE) clustering of 7057 single cells from 2D_CM. Different cell types were assigned with different colors. (G) Heatmap showing the expression pattern of top 10 differential genes for each cluster. Five representative genes for each cluster were listed on the right side. (H) Identification of cell types by the expression of well-known marker genes. Cell counts and the proportion of each cell type (left). Violin plots and *t*-SNE plots showing the expression distributions of well-known maker genes for four cell types (right).

We first generated CMs (2D_CM) from hiPSCs by sequential treatment of CHIR (CHIR99021, GSK3β inhibitor), and IWP2 (WNT inhibitor) (Fig. 1A), using published methods with minor modifications [8, 9]. Cardiac troponin T (cTnT) in day 21 hiPSC-CMs showed a typical striated pattern (Fig. 1B). About 73.9% and 60.5% of cells are positive for cTnT and ACTN2, according to flow cytometry analysis (Fig. 1C). Immunostaining and quantification revealed that 53% and 47% of 2D_CMs expressed MLC2a (highly expressed in atrial and immature ventricular CMs) and MLC2v (highly expressed in mature ventricular CMs), respectively (Fig. 1D). Furthermore, rhythmic Ca^2+^ influx was observed on day 21 2D_CMs (Figs. 1E and Video S1).

ScRNA-seq of day 21 2D_CMs produced 7057 high-quality transcriptomes fall into four distinct clusters (Figs. 1F–H). The largest cell cluster (C0, 72%) highly expressed cardiac marker genes (*MYH6*, *NKX2-5*, and *TNNT2*). Cluster 1 (C1, 19%) enriched canonical SMC marker genes, *ACTA2* (α-SMA), *TAGLN* (SM22-α), *CNN1*, and *MYH11* [10]. Cluster 2 (C2, 8%) was identified as proliferative CMs with high levels of cardiac marker genes and cell cycle genes, such as *TOP2A*, *MKI67*, and *HIST1H4C*. A tiny fraction (C3, 1%) upregulated epithelial markers (*FBLN1*, *KRT8*, and *CLDN4*).

The above results showed that our 2D_CM consisted primarily of CMs (~80%), some SMCs (~19%), and rare epithelial cells (~1%), but few fibroblasts and ECs.

### MCO generation promoted CM maturation

**Figure 2.**
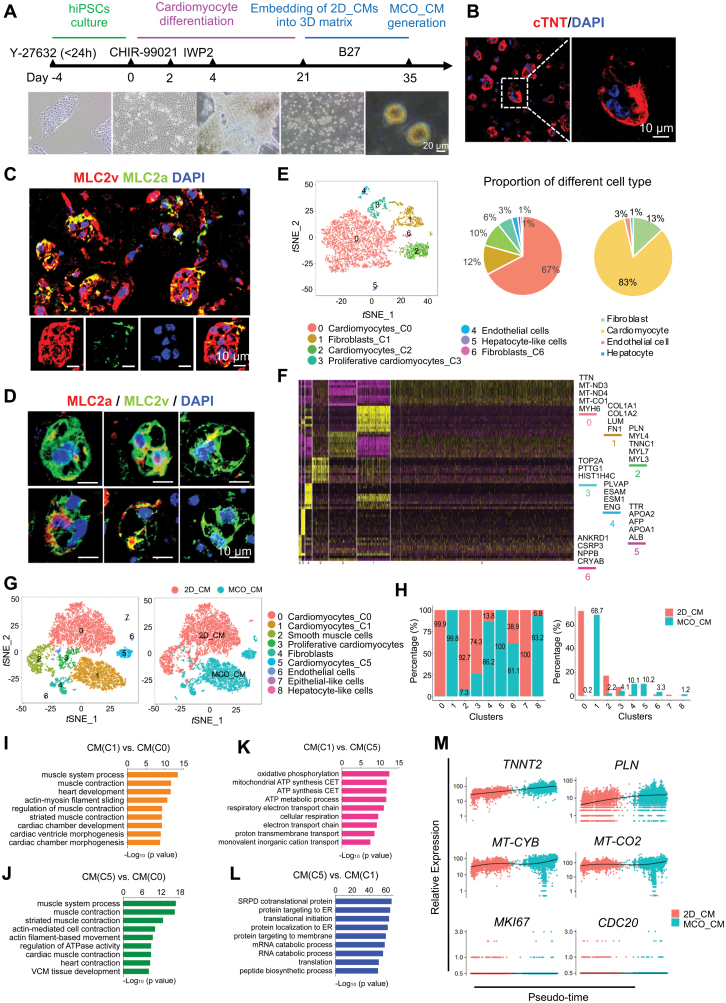
MCO generation and analysis at single-cell resolution.(A) A schematic diagram showing the protocol for preparing 3D scaffold-directed MCO from hiPSCs. The bottom panel corresponds to bright-field images on day −2, day 0, day 21, and day 35 of differentiation. Scale bar, 20 μm. (B) Representative immunofluorescent images of day 35 MCO_CM stained for cTnT. The right panel showing a representative confocal image at high magnification. Scale bar, 10 μm. (C) Representative immunofluorescent images of day 35 MCO_CM stained for MLC2a and MLC2v. Scale bars, 10 μm. (D) Representative confocal images of day 35 MCO_CM stained for MLC2a and MLC2v at high magnification. Scale bars, 10 μm. (E–M). scRNA-seq analysis identifying molecular signatures of MCO_CM. (E) *t*-SNE clustering of 4610 single cells from MCO_CM (left). Cells are marked by cell type and further distinguished with the cluster number for the same cell type. C0–3 and 6: cluster 0–3 and 6. The proportion of each cell type was displayed in pie charts, respectively (right). (F) Heatmap showing the expression pattern of the top 10 differential genes for each cluster. (G) *t*-SNE clustering of 11,651 single cells from 2D_CM and MCO_CM. Cells are marked by cell type and further distinguished with the cluster number for the same cell type. C0, 1, and 5: cluster 0, 1, and 5. (H) The distribution of different samples across different clusters (left) and percentages of different cluster distribution in 2D_CM and MCO_CM, respectively (right). (I–L) GO analysis of upregulated genes among different CM clusters. (M) The expression dynamics of representative genes related to muscle contraction (*TNNT2* and *PLN*), mitochondrial metabolism (*MT-CYB* and *MT-CO2*), and cell cycle (*MKI67* and *CDC20*) in 2D_CM and MCO_CM.

We next seeded day 21 hiPSC-CMs into 3D Collagen-Matrigel and cultured them for 14 days to form MCOs (i.e. MCO_CM) (Fig. 2A). After 3 days, the 3D gel containing CM aggregates contracted significantly (data not shown). On day 35, immunostaining revealed that individual MCO_CM contained highly compact cTnT^+^ CMs (Figs. 2B, S1A and S1B). The size of MCO_CM was 50–200 μm (Figs. S1C and S1D). MCO_CM was mainly composed of MLC2v^+^ cells (Figs. 2C and 2D), suggesting a more mature state. MCO_CM also displayed spontaneously contractile activity as shown by the rhythmic Ca^2+^ influx (Video S2).

We obtained 4610 high-quality single-cell transcriptomes from MCO_CM. Three major cell types and seven distinct cell clusters were identified: CMs (84%), fibroblasts (12%), and ECs (3%) (Figs. 2E, 2F and S1E). Fibroblasts (*COL1A1*, *DDR2*, and *PDGFRA*) and ECs (*APLNR*, *CDH5*, and *PECAM1*) emerged after 3D culture, although they were not in the input hiPSC-CMs (Figs. 2E and 2F). Flow cytometry analysis confirmed the presence of 71.2% cTnT^+^ CMs, 11.4% PDGFRA^+^ fibroblasts, and 4.41% CD31^+^ ECs in MCO_CM (Fig. S1F).

The single-cell analysis also uncovered the effect of 3D culture on CM maturation. Single-cell transcriptomes from 2D_CM and MCO_CM were pooled, and three CM subpopulations could be identified as C0, C1, and C5 (Figs. 2G, 2H and Table S1). C0 in 2D_CM highly expressed *RSPO3*, *ENO1*, and *FGF18*, typically found in early CMs. In contrast, C1 and C5 in MCO_CM highly expressed myosin light chain classes (*MYL4*, *MYL2*, and *MYL3*) and sarcomere contraction genes (*TNNT2*, *TNNC1*, and *TNNI1*), which regulate actin–myosin filament sliding and heart contraction (Figs. 2I and 2J). In addition, C1 in MCO_CM elevated the expression of mitochondrial respiratory chain-related genes (*MT-CYB*, *MT-ND5*, and *MT-CO2*), indicating energy demands for CM maturation (Figs. 2K and 2L). Furthermore, expression dynamics of representative genes along pseudotime trajectory revealed the developmental progression of CMs during MCO generation (Fig. 2M).

The above results demonstrated that CMs cultured in a 3D ECM-rich environment gained molecular profiles towards maturation, including the upregulation of genes needed for increased energy metabolism and contractile force. Besides, fibroblasts and ECs, two important cell types comprising the heart tissue, also emerged upon 3D culture.

### Single-cell analysis of trilineage MCO revealed changes in cell fate and state

**Figure 3.**
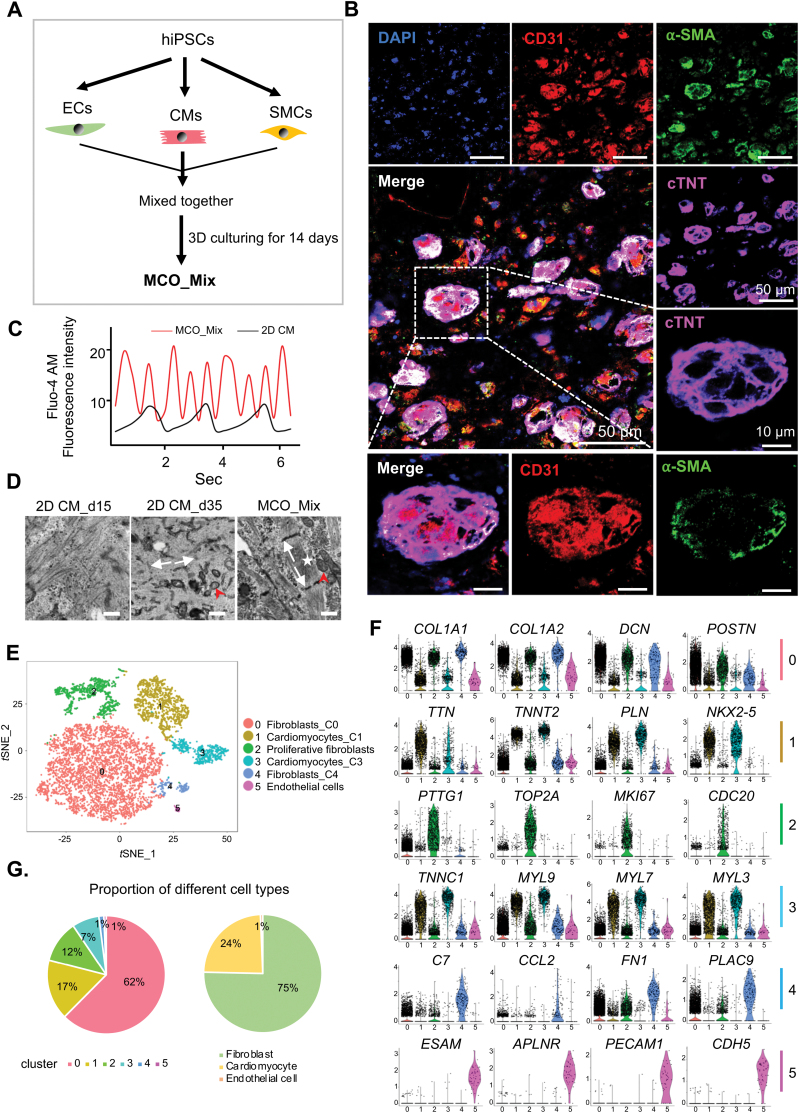
Characterization of trilineage MCO at single-cell resolution.(A) A schematic diagram showing the protocol for generating trilineage MCO (MCO_Mix) from hiPSCs. (B) Representative immunofluorescent images of day 35 MCO_Mix positive for CD31, α-SMA, and cTnT. Scale bars, 50 and 10 μm (zoom-in views of one selected MCO_Mix). (C) Representative calcium flux traces of CMs in 2D_CM and MCO_Mix. The CMs from both 2D_CM and MCO_Mix were cultured *in vitro* for 35 days. (D) Representative transmission electron microscopy (TEM) images showing CM sarcomeres in 2D_CM (left) and MCO_Mix (right). Z-lines (white arrows), M-bands (arrowheads), and mitochondria (red arrows). Scale bars, 500 nm. (E–G) scRNA-seq analysis identifying MCO_Mix populations on day 35 of differentiation. (E) *t*-SNE clustering of 7161 single cells from MCO_Mix. Cells are marked by cell type and further distinguished with the cluster number for the same cell type. C0, 1, 3, and 4: cluster 0, 1, 3, and 4. (F) Violin plots showing the expression distributions of key maker genes across cell types. (G) The proportion of each cell type cluster and the classified population were displayed in pie charts.

Non-CMs play essential roles during cardiac tissue development *in vitro* [11, 12]. Next, we constructed MCO_Mix using hiPSC-derived CMs, ECs and SMCs (Fig. 3A). We first characterized ECs and SMCs using immunostaining and flow cytometry. On differentiation day 8, 51% of the hiPSC-derived ECs were CD144^+^/CD31^+^ (Figs. S2A–C), while more than 95% of hiPSC-SMCs were positive for SM22-α and α-SMA (Figs. S2F–H).

Before constructing MCO_Mix, we also characterize EC and SMC culture at the single-cell level. EC culture contained four major cell types, including epithelial cells (*EPCAM* and *CDH1*), HAND1^+^ mesoderm cells (*HAND1* and *MEST*), ECs (*PLVAP*, *APLN*, and *KDR*), and mesenchymal cells (*COL1A1*, *SNAI2*, and *VIM*) (Figs. S2D and S2E). In comparison, SMC culture could be divided into four clusters such as early SMCs (*ACTA2* and *TAGLN*), late SMCs (*CNN1* and *MYH11*), cardiac mesoderm cells (*SLN*, *RSPO3*, and *HAND2*), and hepatocyte-like cells (Figs. S2I and S2J). Thus, EC and SMC could bring in more non-CM cell types for MCO_Mix generation.

We next mixed CM with EC and SMC at a ratio of 2:1:1 (Fig. 3A). Strikingly, the addition of EC and SMC greatly accelerated the formation of Collagen-Matrigel constructs, which condensed in less than 24 h. In the meantime, cells were self-assembled into compact aggregates. We performed immunostaining of MCO_Mix 14 days after culturing in Collagen-Matrigel and found CD31^+^/α-SMA^+^ cells in most cell aggregates (Fig. 3B). MCO_Mix (14 days) showed strong contractile function, as the intensity and frequency of their Ca^2+^ transient were much higher than that of 2D_CM (35 days) (Fig. 3C). In addition, transmission electron microscopy revealed that MCO_Mix (14 days) also had a more robust sarcomere structure than 15 and 35 days cultured 2D_CM (Fig. 3D). ScRNA-seq analysis showed six transcriptionally distinct clusters within MCO_Mix (Figs. 3E, 3F and S3A). They belong to three major cell types: CMs (24%), fibroblasts (75%), and ECs (1%) (Fig. 3G). Flow cytometry analysis of MCO_Mix showed similar cell ratio: 20.0% cTnT^+^ CMs, 69.3% PDGFRA^+^ fibroblasts, and 4.21% CD31^+^ ECs (Fig. S3B). Thus, during trilineage MCO formation, the proportion of CMs reduced, while the proportion of fibroblasts greatly increased.

### Characterization of CM maturation status in MCO

**Figure 4.**
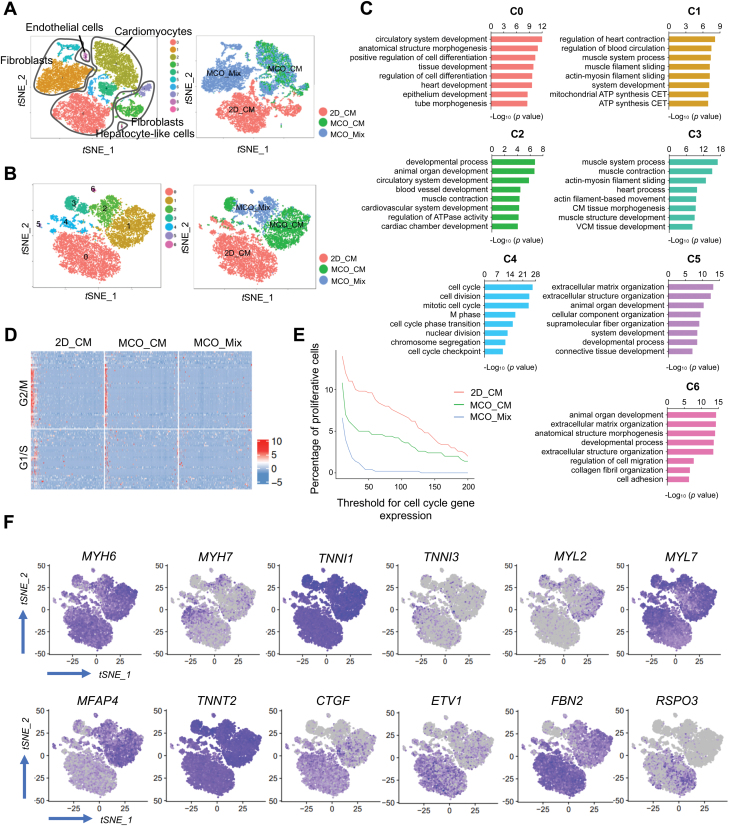
Single-cell map of CM maturation in MCOs.(A) *t*-SNE clustering of 18,795 single cells from 2D_CM, MCO_CM, and MCO_Mix. Four identified cell types were labeled in the *t*-SNE map. (B) *t*-SNE plots showing the second-level clustering of all CMs. (C) Representative enriched GO terms of each subcluster. CET, coupled electron transport; VCM, ventricular cardiac muscle. (D) Heatmap showing *Z* score scaled expression level of cell cycle genes in CMs from 2D_CM, MCO_CM, and MCO_Mix, respectively. (E) The proportion of proliferative cells based on the heatmap of cell cycle gene expression. (F) *t*-SNE plots showing the expression distributions of cardiac maker genes for CMs from 2D_CM, MCO_CM, and MCO_Mix.

To better understand the maturation process after 3D culture with mixed cell types, we extracted CMs from 2D_CM, MCO_CM, and MCO_Mix datasets for comparative analysis (Figs. 4A, 4B, S4A and S4B). Subcluster C0 (CMs from 2D_CM) has high levels of regulatory genes for cardiac fate specification, including *RSPO3*, *ETV1*, and *ANKRD1* [13–15], as well as early CM genes (*CLDN6*, *COL2A1*, and *S100A10*) (Table S2) [16]. On the contrary, subcluster C1 (primarily from MCO_CM) is related to muscle system process and heart contraction, with significant upregulation of mitochondrial metabolism-related genes for energy demands. Subcluster C2 was mainly derived from MCO_Mix, enriched for muscle contraction and blood vessel development (Fig. 4C). Subcluster C3 from both MCO_CM and MCO_Mix was exclusively associated with muscle structure development and actin filament-based movement, a sign of enhanced muscle mechanical features. Subcluster C4 represented actively proliferative CMs with high expression of cell cycle genes and mostly come from 2D_CM. The proportion of proliferative CMs decreased dramatically after MCO generation, down from 10.9% in 2D_CM to 5% in MCO_CM and 1.4% in MCO_Mix (Figs. 4C and 4D). Cell cycle activity is an important feature in evaluating CM maturation. Cell cycle analysis showed that most CMs from MCO_Mix have exited the cell cycle, indicating increased maturity (Figs. 4D and 4E). Subcluster C5 (98.7% of cells from 2D_CM) and C6 (all cells from MCO_Mix) highly expressed ECM genes (Figs. S4C and S4D), which were active both at the early and late stages during heart development [17, 18]. The expression pattern of representative cardiac marker genes indicated increased CM maturation in MCO_CM and MCO_Mix compared to 2D_CM. The expression of *TNNT3*, *MYH7*, *MYL7*, *MFAP4*, and *TNN2* was generally higher in MCO_Mix and MCO_CM, while those of *MYH6*, *TNNI1*, and *MYL2* was lower in 2D_CM (Fig. 4F) [19]. Moreover, genes associated with cardiac progenitor cells and immature CMs, such as *CTGF*, *ETV1*, *FBN2*, and *RSPO3,* were much higher in 2D_CM, suggesting the CMs transit to a more mature state in MCO_Mix (Fig. 4F).

We also reconstructed a pseudotime trajectory of CM maturation from pooled CMs from 2D_CM, MCO_CM, and MCO_Mix (Fig. S5A). There were three bifurcation points in the whole trajectory, giving rise to seven branches (labeled with the lowercase letters a–g) corresponding to CMs at different maturation states (Fig. S5A). Cells in 2D_CM are located to four branches, including pre-branch (a), *PLN*-high late-stage CMs branch (b), mitochondrial ATP metabolism active branch (c), and *RSPO3*-high early-stage CMs (d), indicating that CMs in 2D_CM are highly heterogenous and immature (Figs. S5B and S5C). After MCO generation, most cells in MCO_CM fell into one terminal branch, characterized by dramatically decreased expression of cardiovascular developmental genes and increased expression of genes involved in mitochondrial ATP production. In contrast, cells in MCO_Mix gathered in another terminal with high expression of mature cardiac markers (*PLN*, *MYL2*, and *TNNC1*) (Figs. S5A–C). Thus, the pseudotime trajectory analysis provided a molecular roadmap of CM maturation in an ECM-rich and multicellular microenvironment.

### Dynamic change of fibroblast population in MCO

**Figure 5.**
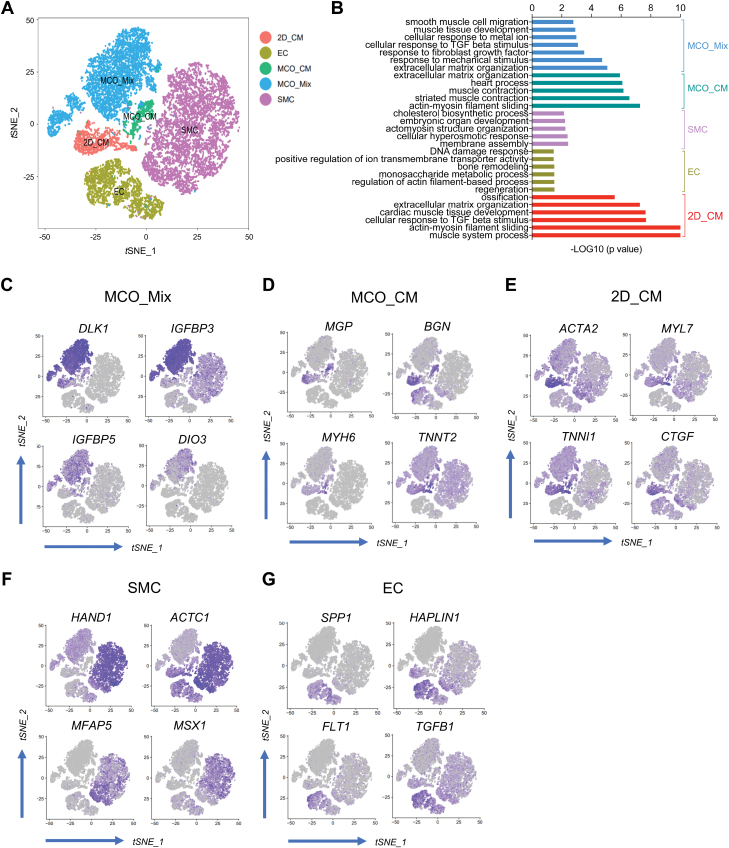
Single-cell map of fibroblasts during MCO formation.(A) *t*-SNE plots showing the second-level clustering of all *PDGFRB*^+^ cells from 2D_CM, EC, SMC, MCO_CM, and MCO_Mix. (B) GO analysis of upregulated genes in fibroblasts from each sample. (C–G) *t*-SNE plots showing the expression distributions of enriched genes in fibroblasts from different groups.

We next examined the characteristics of fibroblast-like cells from different groups. PDGFRβ is a marker for a broad range of mesoderm cells with mesenchymal characteristics, including SMCs, mesoderm progenitors, mesenchymal cells, and fibroblasts of all development stages [20, 21]. Interestingly, *PDGFRB*^+^ cells from 2D_CM, EC, SMC, MCO_CM, and MCO_Mix formed distinct clusters (Fig. 5A). Fibroblasts from MCO_Mix highly expressed genes that are enriched with Gene Ontology (GO) terms such as ECM organization, response to mechanical stimulus, FGF, TGFβ and metal ion, muscle tissue development, etc. (Fig. 5B). Fibroblasts in MCO_CM expressed more muscle contractile-related genes (Fig. 5B). Fibroblasts from 2D_CM and MCO_CM showed obvious cardiac gene signatures (Fig. 5B). On the other side, fibroblasts in EC and SMC culture did not express cardiac genes but were enriched with genes associated with cholesterol metabolism, cellular hyperosmotic response, membrane assembly, and actin filament-based process (Fig. 5B). Thus, the CM-rich microenvironment had a significant influence on fibroblast transcriptome. The fibroblast clusters will likely represent the distinct transcriptional state adapted to different cellular contexts. Notably, two well-known imprinted genes related to growth and differentiation, *DLK1* and *DIO3*, are uniquely highly expressed in MCO_Mix fibroblasts (Fig. 5C). The cell map showing representative differentially expressed genes (DEGs) is listed in Figs. 5C–G, consistent with the enriched GO terms in each cluster.

As fibroblast cells in MCO_Mix came from 2D_CM, EC, and SMC, we used pseudotime analysis to dissect their relationship (Fig. S6A). All *PDGFRB*^+^ cells formed a trajectory structure (Fig. S6A). Fibroblasts from MCO_Mix were located at the right end and expressed all fibroblast marker genes, suggesting they are bona fide fibroblasts (Fig. S6A). Fibroblast cells from EC and SMC (left end) and 2D_CM (middle) shared some early smooth muscle features, notably, higher expression of *ACTA2* and *TAGLN* (Figs. S6A–C). Besides common fibroblast marker genes (*COL1A1*, *PDGFRA*, and *POSTN*), MCO_Mix fibroblasts specifically express *TCF21*, a key transcription factor involved in epicardial epithelial-to-mesenchymal transition and fibroblast fate determination (Figs. S6B and S6C) [22–24]. Pseudotime analysis suggested that after mixing and 3D culture, fibroblast-like cells from 2D_CM, EC, and SMC transformed into a distinct and uniform population of fibroblast cells, where growth and differentiation, and ECM genes were upregulated, but early smooth muscle genes were downregulated (Figs. S6B and S6C).

To rule out the possibility that prolonged culture in 2D can also cause a cell state change, we analyzed scRNA-seq datasets from published studies using a similar differentiation protocol [16, 25]. In Churko *et al*.’s study [16], the expression pattern of smooth muscle genes *ACTA2* and *TAGLN* did not downregulate from differentiation day 14 to 45 (Figs. S7A and S7B). A similar trend was observed in the Friedman *et al*.’s study [25] (Figs. S7C and S7D). No fibroblast population emerged in later differentiation days, suggesting the cell state shift observed in our study is associated with the 3D culture.

Together, these results suggested that the 3D multilineage microenvironment reprogrammed the fibroblast state. As fibroblasts play important roles in promoting CM maturation and heart repair, the increasing proportion of fibroblasts in the MCO_Mix may boost its reparative potential.

### DLK1 signaling induced change in immune-modulatory gene expression in CMs

**Figure 6.**
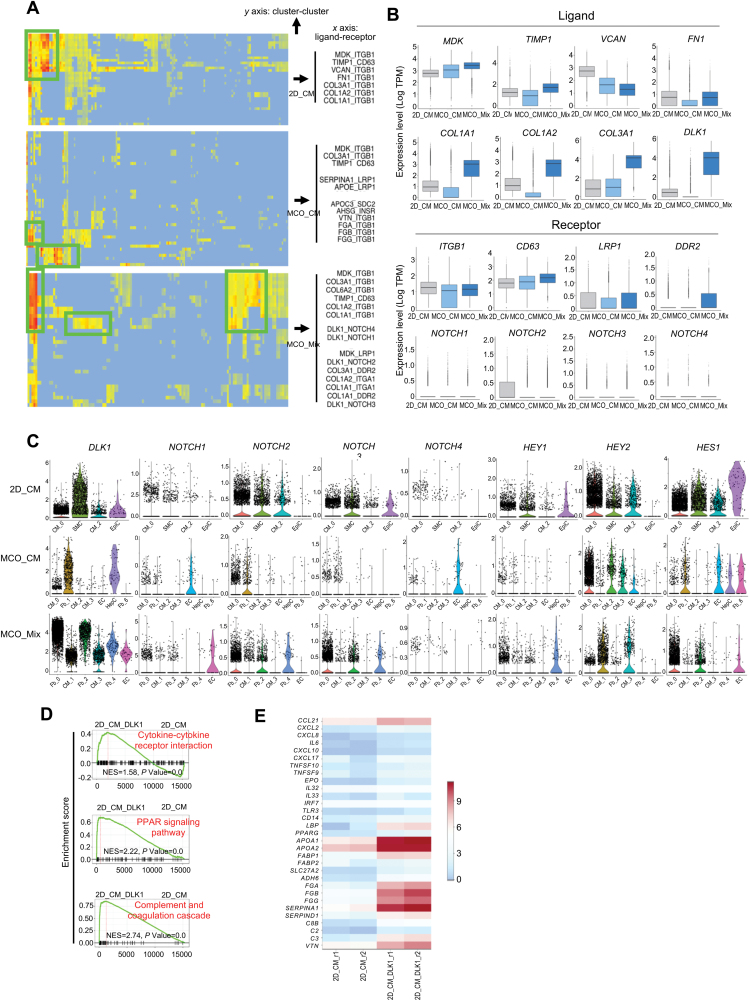
DLK1 signaling regulates immune-modulatory gene expression in CMs.(A) Heatmap showing the mean expression of receptor–ligand pairs among cell clusters (see Materials and methods for expression calculation). (B) Histograms showing the mean expression of selected ligands and receptors in 2D_CM, MCO_CM, and MCO_Mix. (C) Violin plots showing the expression distributions of DLK1, NOTCH receptors (*NOTCH1*, *NOTCH2*, *NOTCH3*, and *NOTCH4*), and NOTCH targets (*HES1*, *HEY1*, and *HEY2*) in 2D_CM, MCO_CM, and MCO_Mix. *X*-axis showing abbreviations for cell type clusters for each sample. 2D_CM: CM_0, SMC, CM_2, and EpiC represent cardiomyocytes from cluster 0, smooth muscle cells, proliferative cardiomyocytes from cluster 2, and epithelial-like cells, respectively. MCO_CM: CM_0, Fb_1, CM_2, CM_3, EC, HepC, and Fb_6 represent cardiomyocytes from cluster 0, fibroblasts, cardiomyocytes from cluster 2, proliferative cardiomyocytes from cluster 3, endothelial cells, hepatocyte-like cells, and fibroblast cells from cluster 6, respectively. MCO_Mix: Fb_0, Fb_2, and Fb_4 represent fibroblasts from cluster 0, 2, and 4, respectively; CM_1 and CM_3 represent cardiomyocytes from cluster 1 and 3; EC represents endothelial cells. (D) GSEA results showing representative enriched signaling pathways between 2D_CM_DLK1 and 2D_CM. 2D_CM_DLK1 and 2D_CM represent day 21 hiPSC-CMs with and without sDLK1 treatment for 14 days, respectively. (E) Heatmap comparing bulk expression of representative marker genes for cytokine–cytokine receptor interaction, PPAR signaling pathway, and complement and coagulation cascade in 2D_CM vs. 2D_CM_DLK1.

To understand the cell interactions involved in MCO development, we performed a receptor–ligand pairing analysis of 2D_CM, MCO_CM, and MCO_Mix. Integrin receptor–ligand pairs, which mediate cell–ECM interaction, cell migration, and activation, were prominent compared to other cell adhesion molecules, such as the interactions between secreted growth factor (*MDK*) as well as ECM-related genes (*TIMP1*, *COL1A1*, *COL1A2*, and *COL3A1*) and their receptor genes (*ITGB1*, *CD63*, *LRP1*, and *DDR2*) (Figs. 6A and 6B). Notably, *DLK1* was highly upregulated only in fibroblasts from MCO_Mix (Figs. 6A–C). Other receptors and ligands of the NOTCH signaling pathway remained at relatively low levels in all three groups (Fig. 6B). NOTCH pathways target genes *HES1* and *HEY2* were elevated in fibroblasts and ECs and CMs of MCO_Mix, respectively (Fig. 6C), suggesting differential NOTCH signaling activation in these cell populations.

Next, we asked whether supplement DLK1 could have a beneficial impact on 2D_CMs. We treated CM with soluble DLK1 (sDLK1). Flow cytometry analysis showed that sDLK1 treatment did not affect CM differentiation efficiency (data not shown). Bulk RNA profiling was then performed to analyze the changes in global gene expression. Gene set enrichment analysis (GSEA) coupled with the Kyoto Encyclopedia of Genes and Genomes (KEGG) pathway analysis showed that sDLK1 treatment enhanced the expression of genes related to “cytokine-cytokine receptor interaction” (e.g. *CCL21*, *CXCL2*, and *IL32*), “PPAR signaling pathway” (e.g. *APOA1/2*, *FABP1/2*, and *SLC27A2*), “complement and coagulation and cascade” (e.g. *FGA*, *FGG*, and *C8B*), and “Toll-like receptor signaling pathway” (e.g. *TLR3*, *CXCL8*, *CXCL10*, and *IL6*) (Figs. 6D, 6E, S8A and S8B) [26]. GO terms associated with inflammatory regulation and extracellular structure organization became enriched after sDLK1 treatment (Figs. S8C and S8D).

Altogether, these data suggest that the *DLK1*^high^ fibroblasts in the MCO_Mix could enhance the immune-modulatory and reparatory potential of MCO.

### MCO transplantation significantly improved heart function after MI

**Figure 7.**
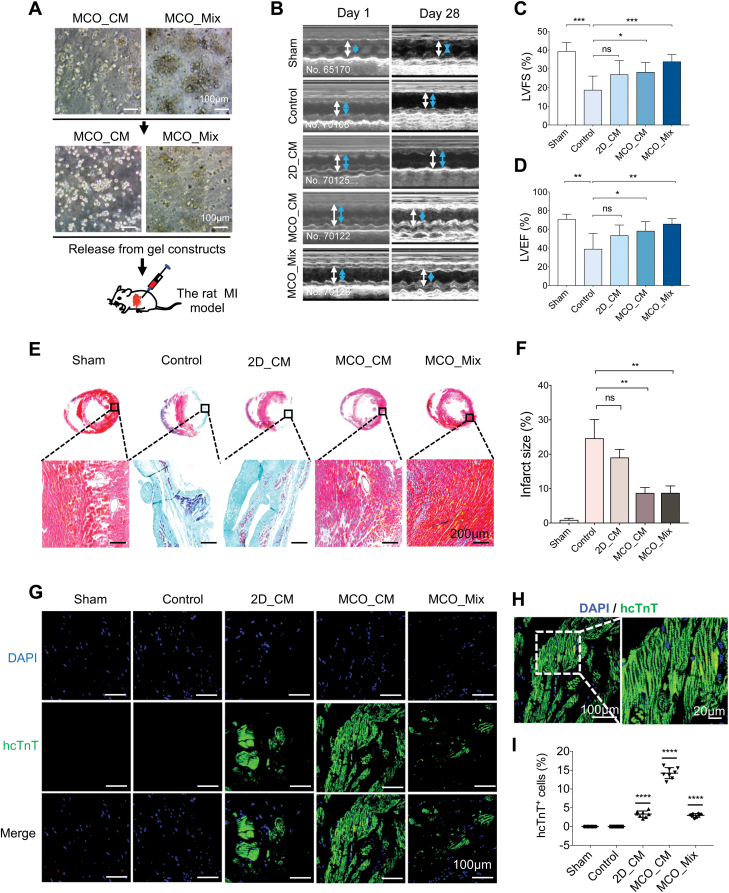
Transplantation of MCO to a rat model of MI and evaluation of heart repair.(A) Representative bright-field images showing MCO_CM and MCO_Mix before and after being released from Collagen-Matrigel gel constructs prepared for transplantation into the rat MI model. Scale bars, 100 µm. (B) Representative M-mode echocardiographic images in Sham, Control, 2D_CM, MCO_CM, and MCO_Mix groups on day 1 and day 28 post-transplantation. Arrows indicate LV end-diastolic dimensions (white) and end-systolic dimensions (blue). (C, D) Quantitative cardiac function analysis of LVFS and LVEF 28 days after MI procedure and cell transplantation. Data were mean ± SD. Sham (*n* = 6), Control (*n* = 6), 2D_CM (*n* = 8), MCO_CM (*n* = 8), and MCO_Mix (*n* = 8). *P < 0.05, **P < 0.005, ***P < 0.001, *t*-test. (E) Representative images of rat heart sections stained with Masson’s trichrome on day 28 post-transplantation. Scale bars, 200 µm. (F) Quantitative analysis of relative fibrosis area from Masson’s trichrome staining data. Data expressed as mean ± SD. (G) Representative immunofluorescent images of rat heart sections stained for hNA (red) and hcTnT (green) on day 28 post-transplantation. Scale bars, 100 µm. (H) Representative confocal images of MCO_CM group and zoom-in views of hcTnT^+^ cells showing evident striated pattern with green fluorescence. Scale bars, 100 µm (top) and 20 µm (bottom). (I) Quantification of hcTnT^+^ cells per field of view in G from six rats for each group, eight random fields of view for each group; ****P < 0.0001, unpaired *t*-test.

To validate MCOs *in vivo* therapeutic potential, we performed transplantation in a rat model of MI, in which the left anterior descending coronary artery was permanently ligated. MCO_CM and MCO_Mix were released from the 3D gel and injected through a 28-gauge needle into the border zone around the infarcted area (Figs. 7A and S9A). The experimental design is shown in Fig. S9B.

Echocardiographic examination showed that 24 h after MI procedure and cell transplantation, both diastolic and systolic cardiac function decreased significantly in MI rats compared to Sham group (Fig. 7B). Twenty-eight days after transplantation, left ventricle ejection fraction (LVEF; 38.8% ± 16.8%) and left ventricle fractional shortening (LVFS; 18.6% ± 7.5%) were substantially reduced in Control MI rats compared to Sham group’s LVEF (70.8% ± 5.5%, *P* < 0.05) and LVFS (39.3% ± 4.8%, *P* < 0.05) (Figs. 7B–D, S9C). Transplantation of MCO_Mix increased LVEF (65.6% ± 5.9%, *P* < .005) and LVFS (33.8% ± 3.9%, *P* < 0.001) the most, more than transplantation of MCO_CM, LVEF (58.2% ± 10.2%, *P* < 0.05) and LVFS (28.2% ± 5.3%, *P* < 0.05) (Figs. 7B–D, S9C). In contrast, transplanted 2D_CM did not significantly improve LVEF (53.5% ± 11.2%, *P* < 0.05) and LVFS (26.9% ± 7.5%, *P* < 0.05) compared to the control group (Figs. 7B–D).

Twenty-eight days after MCO transplantation, the hearts were harvested for histological analysis. Masson’s trichrome staining revealed that transplantation of MCO significantly reduced the infracted size compared with the Control group (*P* < .005) (Figs. 7E and 7F). In contrast, transplantation of 2D_CM had only a modest effect on reducing collagen deposition (light blue staining) (*P* = .185 vs. Control). Immunostaining revealed areas strongly positive for human-specific cTnT (hcTnT), confirming that human CMs in 2D_CM and MCO survived in the rat heart for 28 days after transplantation (Figs. 7G and 7H). Quantification of hcTnT^+^ cells in the MI hearts revealed that MCO_CM had about three times more human CM survival than MCO_Mix (Fig. 7I), roughly consistent with 83% and 24% CMs in MCO_CM and MCO_Mix from single-cell analysis (Figs. 2E and 3G). These results suggested that the DLK1^+^ fibroblast cells in the MCO_Mix may significantly contribute to the regeneration process.

## Discussion

**Figure 8.**
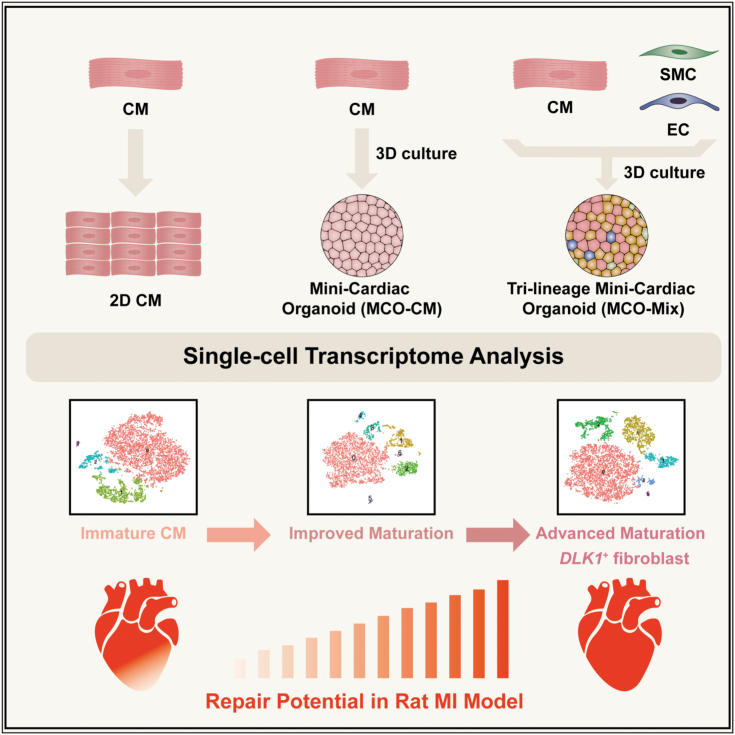
Model of cell state and fate change upon 3D multilineage cardiac organoid formation and the effect on MI repair. The 3D matrix-rich microenvironment significantly enhanced the maturation of CMs. DLK1^+^ fibroblasts emerged upon multilineage cell interaction and exhibited potent regenerative potential in rat MI models.

In this study, we generated cardiac organoids consisting of hiPSC-derived CM, EC, and SMC in 3D ECM-rich gel and employed scRNA-seq to depict a detailed cellular and molecular roadmap of cell fate and state change in a multilineage cardiac organoid context. We also showed that cardiac organoids made from multiple cell types gave the best result in transplantation assays to repair the ischemic rat heart (Fig. 8).

Many studies have shown that PSC-derived CMs were in an immature state [5, 11, 27–29]. 3D culture and ECs, SMCs, and fibroblasts have been used to mimic the *in vivo* microenvironment and the complex intercellular contacts to promote CM maturation [3, 5, 27, 30]. Our results provided an in-depth single-cell perspective of this process. CMs in MCO_CM and MCO_Mix showed coordinated upregulation of mature cardiac structural protein genes, metabolic and energy consumption genes, ECM genes and downregulation of active cell cycle genes. CMs with active cell cycle gene profile decreased from 10.9% in 2D_CM to 5% in MCO_CM and only 1.4% in MCO_Mix. These molecular changes corroborated with the characteristics during structural and physiological maturation described in a recent study by Giacomelli *et al*., which investigated cardiac microtissue made from hPSC-derived CM, EC, and fibroblasts [27].

Cardiac fibroblasts play pivotal roles in CM maturation, heart development, and regeneration *in vivo* and *in vitro* [22, 27, 31]. In our study, the pseudotime trajectory showed fibroblast population in the MCO_Mix is relatively uniform (Fig. 6). Therefore, heterogeneous cell clusters within the input cell population carrying mesenchymal features transformed into fibroblasts with similar molecular signatures. The fibroblasts in the MCO_Mix all upregulated several typical genes, including *TCF21* and *PDGFRA*, consistent with the fibroblast marker genes in other studies [27].

Another interesting feature of fibroblast in MCO_Mix is the significant upregulation of *DLK1*. The anti-fibrosis properties of Dlk1 have been demonstrated via *Dlk1*-knockout mice [32]. *DLK1* is highly expressed in progenitor cells during early tissue development and regeneration [33, 34]. Fibroblasts function as an essential regulator during MI [35–37]. So, the presence of DLK1^high^ fibroblasts in the MCO_Mix might help to prevent pathological cardiac remodeling. The transplantation of MCO_Mix into infarcted rat hearts showed a significant reparatory effect in terms of LVFS and LVEF parameters (Fig. 7B). The infarct size was similar for MCO_CM and MCO_Mix engrafted groups despite more hcTnT-positive areas in MCO_CM-transplanted hearts, suggesting that the fibroblasts in the MCO_Mix might be responsible for improved angiogenesis, reduced cell death, inflammation, and fibrosis. These beneficial effects are in agreement with the transcriptome changes induced by sDLK1. sDLK1-treated 2D_CM upregulated GO terms associated with regulation of inflammatory response, extracellular structure organization, and regulation of response to wounding, which corroborated the immunomodulatory actions of DLK1 reported before [38, 39]. The cytokines upregulated by sDLK1 include CXCL10, which has been shown to prevent premature angiogenesis and fibrous tissue deposition [40]. IL6, IL32, and IL33 played dual modulatory roles in inflammatory conditions and were involved in wound healing [41]. Future research investigating the cellular and molecular change in both engrafted cells and host cells in the infarcted hearts will help elucidate how DLK1^high^ fibroblast cells from MCO_Mix repair and regenerate the heart.

Our study provided a single-cell resolution map of 3D cultured multilineage cardiac organoid, which significantly improved our understanding of cell fate, state change, and underlying gene regulatory networks. We demonstrated that DLK1^+^ fibroblasts emerged upon multilineage cell interaction and exhibited potent regenerative potential towards the infarcted heart *in vivo* (Fig. 8). These findings offer new strategic clues to promote hiPSC-derived CM maturation *in vitro* and heart repair *in vivo*.

## Research limitations

This study also has several limitations. In the MCO_CM and MCO_Mix, cells are mixed and cultured in a 3D ECM-rich environment. Many studies showed that tissue engineering approaches or additional training by applying mechanical force or electrical stimulation could promote the maturation of hiPSC-derived CMs [28]. More sophisticated tissue engineering may be required to improve the maturation of multilineage cardiac organoids further. In addition, although cell transplantation to a rat MI model demonstrated proof-of-concept results, small-animal models do not recapitulate human MI and heart regeneration very well. Preclinical models using nonhuman primates or large animals (swine, dogs, and others) should be used to evaluate the benefits of different cell grafts.

## Materials and methods

### Human iPSC culture

The human iPSCs used in our study were provided by J. Na and F. Duan (Tsinghua University, Center for Stem Cell Biology and Regenerative Medicine). They were generated from umbilical cord blood CD34^+^ cells using the CytoTune™-iPS Reprogramming Kit (Invitrogen) according to the manufacturer’s instructions [42]. For feeder-free culture, hiPSCs were maintained on Matrigel (BD Biosciences)-coated plates (Corning) in TeSR-E8 medium (STEMCELL Technologies).

### Human iPSC cardiac differentiation

Differentiation towards CM lineage was induced using small molecules to modulate the WNT signaling pathway, optimized according to a small molecule-based mono-layer method as previously described [8]. Briefly, undifferentiated hiPSCs cultured in TeSR-E8 medium were digested into single cells by Accutase (Millipore) and plated onto Matrigel-coated culture dishes at a density of 2 × 10^4^ cells/cm^2^ in TeSR-E8 medium with 10 μM ROCK inhibitor (Y27632, Selleck). After 1 day, Y27632 was withdrawn from the medium, and cells were cultured in TeSR-E8 medium for another 3 days. At day 0, cells were treated with 4 μM CHIR99021 (Selleck) in the cardiac differentiation medium, consisting of RPMI 1640 and B27 minus insulin. After 2 days, the medium was changed with 5 μM IWP2 (Selleck) treatment for another 2 days. At day 4, the cells were continuously cultured in the cardiac differentiation medium for another 4 days, which was changed every other day. Contracting cells were seen from day 8 to day 10. After 8 days, the cells were transferred to the medium for maintaining hiPSC-CMs, consisting of RPMI 1640 and B27 (RPMI/B27), and the medium was changed every other day.

ECs and SMCs were differentiated from hiPSCs according to our previously published protocols [43, 44]. Briefly, undifferentiated hiPSCs were digested into single cells by Accutase and plated onto Matrigel-coated culture dishes at a density of 1 × 10^4^ cells/cm^2^ in TeSR-E8 medium with 10 μM Y27632 for 1 day. Cells then were cultured in TeSR-E8 medium for another 1 day. On day 0, the medium was changed to AATS supplemented with 2 μM CHIR99021 for another 3 days. In differentiation stage II, cells were replated onto Matrigel-coated dishes at a density of 4 × 10^4^ cells/cm^2^ in AATS stimulated with 50 ng/mL VEGF165 plus 10 ng ng/mL FGF2 (for EC differentiation) and 10 ng/mL PDGF-BB plus 2 ng/mL TGF-β1 (for SMC differentiation) for 5 days.

### Human iPSC-derived MCO generation

The casting molds were prepared by a method described previously [45]. Then, 1.0 × 10^7^ hiPSC_CMs or a mixture of 5.0 × 10^7^ hiPSC-CMs, 2.5 × 10^7^ hiPSC-ECs, and 2.5 × 10^7^ hiPSC-SMCs were resuspended by 0.5 mL concentrated culture medium (2× H-DMEM, 100 U/mL penicillin, and 100 mg/mL streptomycin). About 0.5 mL liquid collagen type I (1.5 mg/mL) prepared from rat tails, supplemented with 20% Matrigel (BD Biosciences), was mixed with above-mentioned cell suspension; pH was neutralized by titration with 0.1 M NaOH to 7.2. The reconstitution mix was pipetted into 12-well plate casting molds and incubated for 45–60 min at 37°C and 5% CO_2_ to allow hardening. Thereafter, 1.5 mL culture medium RPMI/B27 was added to each well. The culture medium was replaced by fresh medium after 24 h, and then was changed every other day. The MCOs were formed within 3D gel constructs and maintained for 14 days. The MCOs were released by collagenase IV (Thermo) and collected to evaluate the structure and function.

### Immunofluorescence

Both differentiated cells and MCOs were fixed with 4% paraformaldehyde, permeabilized in 0.5% Triton X-100 (Sigma), blocked in 10% normal goat serum (ZSGB-BIO), and then incubated with primary antibodies against cTnT (1:200, R&D), MLC2v (1:200, Proteintech), MLC2a (1:200, Synaptic Systems), CD31(1:100, Santa Cruz), and CD144, SM22-α, α-SMA, human nuclei antibody (hNA), and hcTnT (1:200, Abcam) at 4°C overnight. Signals were visualized with DyLight 488-, 550- or 633-conjugated secondary antibodies (1:500, Thermo) for 1 h at room temperature. DAPI (Sigma) was used to label nuclei. Images were captured using Nikon A1R confocal microscope and analyzed with NIS Elements analysis software.

### Flow cytometry

Cells were dissociated with TrypLE™ (Thermo) and filtered through a 70-μm cell strainer (BD Biosciences) to get a single cell suspension. For CM and SMC characterization, single cells were fixed with 4% paraformaldehyde, permeabilized in 0.1% Triton X-100, and processed with FACS washing buffer (PBS with 5% fetal calf serum and 2.5 mM EDTA). Cells were stained with primary antibodies (cTnT, 1:100; ACTN2, 1:100, Abcam; SM22-α, 1:100) for 1 h at room temperature and labeled with DyLight 488-conjugated secondary antibodies (1:100) for 30 min at room temperature. For EC characterization, single cells were incubated with fluorophore-labeled primary antibodies (CD31, 1:20, Miltenyi Biotec; CD144, 1:20, Miltenyi Biotec) for 20 min at 4°C, followed by FACS washing buffer processing. Cells were analyzed using a FACS Caliber flow cytometer (BD Biosciences), and data were processed using FlowJo 10.4 software (TreeStar).

### Calcium transient analysis

Cells were cultured in 24-well plates and 10 μM Fluo-4 AM (Thermo) was added to RPMI/B27 medium to visualize calcium signals according to standard procedures. Time-lapse fluorescence images of calcium flux were recorded using an Opera Phenix HCS System (PerkinElmer). The fluorescence intensity changes over time were quantified via ImageJ software. Beating areas [region of interest (ROI)] were randomly selected. Each frame was normalized to a background region to give a baseline-corrected value in fluorescence intensity. The average baseline-corrected Fluo-4 AM fluorescence intensity of ROI was quantified for each frame and plotted over time.

### ScRNA-seq library preparation and data preprocessing

All samples were prepared at 1 × 10^6^ cells/mL in PBS. Live/dead assay showed around 90% of the cells were viable. Then, single-cell suspensions were immediately loaded on Chromium Single Cell Controller (10× Genomics), and cDNA and subsequent sequencing libraries were generated using Single Cell 3ʹ v2 chemistry (10× genomics) according to the manufacturer’s protocol. All libraries were sequenced using the HiSeq X Ten system with one sequencing lane per sample. The quality of raw sequencing data was checked using FastQC, and single-cell expression matrix was generated following the cellranger count pipeline (v2.0.1). For integrated analysis of multiple samples, cellranger aggr (v2.0.1) was used to normalize samples to equal read depth. The resulting UMI (unique molecular identifier) matrices from the cellranger pipeline were used in the following analysis.

### Cell clustering and identification of DEGs

Cell type identification was performed using R package Seurat (v2.3.4). Briefly, cells were filtered using three criteria: number of expressed genes, number of UMI count, and percentage of mitochondrial genes in each cell. Cells with any of these metrics fall out of 2 SD of all cells are discarded. Next, highly variable genes were identified and these genes were used to perform principal component analysis. Ten principal components were used to perform cell clustering using FindClusters function in Seurat with resolution parameter set to 0.2. DEGs were identified using FindAllMarkers function comparing each cell cluster against the rest of all cells. Top DEGs were used to generate heatmaps showing marker gene expression for cell clusters.

### Cell cycle analysis

Cell cycle analyses were performed using the CellCycleScoring function in the Seurat package with a previously curated set of G1/S and G2/M genes [46]. We evaluated the cell cycle status of each cell according to the average expression of these two cell cycle gene sets. We tested various thresholds to identify proliferative cells and found that the difference in cell cycle activities between cell clusters is robust to changes in the threshold.

### Pseudotime analysis

Pseudotime analyses were performed using Monocle 2. Only cells with similar identities were selected as input data to avoid erroneous branching into other cell types. The Top 2000 most significantly DEGs were used for ordering cells in pseudotime. BEAM function was used to identify genes with changing expression along pseudotime.

### Receptor–ligand analysis

Receptor–ligand analyses were performed using iTALK package. A connection between two cell clusters were considered valid if mean ligand UMI expression is higher than 0.93 and mean ligand UMI expression is higher than 0.33. We tested many varying thresholds and found they have minimal impact on the overall connection atlas. Interaction maps were generated using the iGraph package.

### Bulk RNA-seq

RNA-seq samples were harvested from hiPSC-CMs (2D_CM, day 21), hiPSC-ECs (EC, day 8), hiPSC-SMCs (SMC, day 8), and 2D_CM with/without soluble DLK1 protein (recombinant human Pref-1/DLK1/FA1, R&D Systems) treatment. Total RNA was extracted with Trizol, 100 ng of RNA was converted to cDNA, and cDNA libraries were prepared using the Smart-seq2 protocol [47]. RNA sequencing was performed using the Illumina HiSeq X Ten system.

### MI and MCO transplantation

An acute MI model was produced by ligation of the left coronary artery (6-0 Prolene suture) in sodium pentobarbital-anesthetized (30 mg/kg) male Sprague-Dawley rats (250 ± 10 g) as described previously [48]. Ten minutes later, the infarct region was confirmed by myocardial blanching. Injections were made along the border zone of the infarcted area at three sites (below the left atrium, in the middle portion of the LV, and at the apex) with a total volume of 100 μL using a 28-gauge needle.

Rats were randomized into five groups: (i) Sham group (*n* = 6), underwent thoracotomy and cardiac exposure without coronary ligation; (ii) Control group (*n* = 6), occlusion with PBS; (iii) 2D_CM group (*n* = 8), occlusion with 1 × 10^7^ hiPSC-CMs; (iv) MCO_CM group (*n* = 8), occlusion with MCOs derived from 1 × 10^7^ hiPSC-CMs; and (v) MCO_Mix (*n* = 8), occlusion with MCOs derived from 2.5 × 10^6^ hiPSC-ECs, 2.5 × 10^6^ hiPSC-SMCs, and 5.0 × 10^6^ hiPSC-CMs.

### Evaluation of the physiological function of the heart

Echocardiographic studies were performed 1 day and 28 days after transplantation using a Vevo 2100 system (VisualSonic, Toronto, Canada). Left ventricle end-diastolic dimension (LVEDD) and left ventricle end-systolic dimension (LVESD) were measured with M-mode tracings. Also, LVEF and LVFS were calculated as follows: LVEF (%) = [(LVEDD^3^ – LVESD^3^)/LVEDD^3^] × 100; LVFS (%) = [(LVEDD − LVESD)/LVEDD] × 100.

### Histology analysis

Rat hearts were harvested at 28 days after cell transplantation. They were fixed in 4% paraformaldehyde and paraffin embedded for histological assessment. Masson’s trichrome staining was performed to evaluate fibrosis area using Image J software. Images were acquired with a slide scanner microscope Axio Scan Z1 (Carl Zeiss, Gottingen, Germany) and ZEN software (Carl Zeiss).

### Compliance with ethical guidelines

This article does not contain any studies with human subjects performed by any of the authors. All rat experiments were conducted in accordance with the Guide for the Care and Use of Animals for Research Purposes and approved by the Institutional Animal Care and Use Committee (IACUC) of the Chinese Academy of Military Medical Science.

### Statistical analysis

Quantitative data are expressed as mean ± SD. The statistical significance was determined using a Student’s *t*-test (two-tail) for two groups or one-way analysis of variance for multiple groups. A value of *P* < .05 was considered statistically significant.

### Data availability

Raw RNA-seq and single-cell RNA-seq data are publicly available at the Gene Expression Omnibus (GEO) with accession number GSE166462. All other relevant data are available from the corresponding author upon reasonable request.

## Supplementary Material

lnac002_suppl_Supplementary_Figures

lnac002_suppl_Supplementary_Table_S1

lnac002_suppl_Supplementary_Table_S2

lnac002_suppl_Supplementary_Video_S1

lnac002_suppl_Supplementary_Video_S2
