# Supplementary material for: Single-cell atlas of multilineage cardiac organoids derived from human induced pluripotent stem cells
Source: Life Med. 2022 Jun 14;1(2):179–95. doi: 10.1093/lifemedi/lnac002 (PMC11748996; doi:10.1093/lifemedi/lnac002)
Supplement: lnac002_suppl_Supplementary_Figures [file lnac002_suppl_Supplementary_Figures.docx]

**Single-cell atlas of multilineage cardiac organoid derived from
human induced pluripotent stem cells**

**Supplementary Figures**

**Supplementary Figure 1. Characterization of MCO_CM, related to Figures 1 and 2.** **A.** Representative immunofluorescent images of MCO_CM stained with cTnT, viewed at different magnifications. Scale bars, 100 μm (top) and 10 μm (bottom). **B.** Representative views of single MCO_CM stained with cTnT. Scale bars, 10 μm. **C.** Phase-contrast image of MCO_CM released from 3D Collagen-Matrigel constructs. Scale bars, 100 μm. **D.** Distribution of MCO_CM diameter sizes. **E.** Marker genes for each subpopulation of MCO_CM. The number of cells counted in each subpopulation and the ratio of each subpopulation to the whole population (left). Violin plots and *t*-SNE plots showing the expression distributions of specific maker genes across the whole population (right). **F.** Representative flow cytometric analysis showing expression levels of CD31, PDGFRA, and cTnT in MCO_CM.

**Supplementary Figure 2. Single-cell RNA-seq analysis of hiPSC-derived ECs and SMCs, related to Figure 3.** **A.** A schematic diagram showing the protocol for monolayer-based endothelial differentiation from hiPSCs. **B**. Representative immunofluorescent images showing day 8 hiPSC-ECs positive for CD31 and CD144. Scale bars, 50 μm. **C.** Representative flow cytometric analysis showing expression levels of CD31 (PECAM1) and CD144 (VE-cadherin) in hiPSC-derived endothelial cells (hiPSC-ECs) on day 8 of differentiation. **D.** *t*-SNE plots clustering of 6,014 single cells from EC scRNA-seq data. Four cell type clusters are labeled in the *t*-SNE map, including epithelial cells, HAND1^+^ mesoderm cells, endothelial cells, and mesenchymal cells. **E.** A heatmap showing the expression pattern of top 10 differential genes for each cluster of EC. **F.** A schematic diagram showing the protocol for monolayer-based smooth muscle differentiation from hiPSCs. **G.** Representative immunofluorescent images showing day 8 hiPSC-SMCs positive for SM22-α and α-SMA. Scale bars, 50 μm. **H.** Representative flow cytometric analysis showing the expression level of SM22-α (TAGLN) in hiPSC-derived smooth muscle cells (hiPSC-SMCs) on day 8 of differentiation. **I.** *t*-SNE plots clustering of 7,555 single cells from SMC scRNA-seq data. Four cell type clusters are labeled in the *t*-SNE map, including early smooth muscle cells, late smooth muscle cells, cardiac mesoderm cells, and hepatocyte-like cells. **J.** A heatmap showing the expression pattern of the top 10 differential genes for each cluster of SMC.

**Supplementary Figure 3. Single-cell RNA-seq analysis of MCO_Mix, related to Figure 3.** **A.** A heatmap showing the expression pattern of the top 10 differential genes for each cluster. **B.** Representative flow cytometric analysis showing expression levels of CD31, PDGFRA, and cTnT in MCO_mix sample.

**Supplementary Figure 4. Integrated analysis of single-cell RNA-seq datasets including 2D_CM, MCO_CM, and MCO_Mix, related to Figure 4.** **A.** Integrated analysis of 2D_CM, MCO_CM, and MCO_Mix single-cell RNA-seq datasets by *cellranger aggr* pipeline and violin plots showing the expression distributions of the cardiac maker (top), fibroblast marker (middle), and cell cycle-related (bottom) genes of each cluster across the whole population. **B.** A heatmap showing the expression pattern of the top 10 differential genes for each cluster. **C.** The proportion of each sample among each cluster after performing second-level clustering of all cardiomyocytes. **D.** The distribution of each sample among each cluster after performing second-level clustering of all cardiomyocytes.

**Supplementary Figure 5. Pseudotime analysis of all cardiomyocytes from 2D_CM, MCO_CM, and MCO_Mix by Monocle 2, related to Figure 4.** **A.** Monocle analysis showing the ordering of cardiomyocytes in pseudotime. Cells were colored according to different samples (middle) and clusters (right). Arrows indicated the direction of pseudotime trajectories across the cardiomyocyte maturing process. **B.** Heatmap showing the gene expression dynamics of cardiomyocytes during MCO generation. Genes (row) were clustered, and cells (column) were ordered according to the pseudotime trajectory. Gene cluster I-VI (labeled in boxes) were selected for further analysis. **C.** The expression dynamics of representative marker genes of muscle contraction, early CM development, and ATP metabolic process. **D.** Gene expression pattern of two branches during hiPSC-CM differentiation, from pre-branch (a) to branch (b) and branch (c). Representative marker genes for specific clusters are shown in the right boxes. **E.** Gene expression pattern of two branches during MCO formation, from pre-branch (c) to branch (d) and branch (e). Representative marker genes for specific clusters are shown in the right boxes.

**Supplementary Figure 6. Single-cell analysis of fibroblast during MCO_Mix formation, related to Figure 5.** **A.** Trajectory reconstruction showing the pseudotime development of fibroblasts. *t*-SNE plots showing the second-level clustering of all PDGFRB-expressing cells from 2D_CM, EC, SMC, MCO_CM and MCO_Mix (left), ordered in pseudotime along the trajectory (right). **B.** Expression distributions of early smooth muscle genes (*ACTA2* and *TAGLN*), ECM genes (*COL1A1*, *COL1A2*, and *COL3A1*), fibroblast marker genes (*DDR2*, *DCN*, *PDGFRA*, and *POSTN*), and cardiac-specific transcription factor (*TCF21*) in PDGFRB-expressing cells according to pseudotime orders. **C.** The expression dynamics of early smooth muscle genes (*ACTA2* and *TAGLN*), ECM genes (*COL1A1*, *COL1A2*, and *COL3A1*), fibroblast marker genes (*DDR2*, *DCN*, *PDGFRA*, and *POSTN*), and cardiac-specific transcription factor (*TCF21*) in PDGFRB-expressing cells.

**Supplementary Figure 7. Analysis of cell state and fate in hiPSC-CMs after prolonged 2D culture, related to Figure 4.** **A.** *t*-SNE map of day 14 and day 45 hiPSC-CM culture. ScRNA-seq datasets were from Churko et al. (Churko et al., 2018) **B.** *t*-SNE maps and violin plots showing distributions of marker genes on day 14 and 45 hiPSC-CM culture. **C.** *t*-SNE map of day 15 and day 30 hiPSC-CM culture. ScRNA-seq datasets were from Friedman et al. (Friedman et al., 2018). **D.** *t*-SNE maps and violin plots showing the expression pattern of marker genes on day 15 and 30, respectively.

**Supplementary Figure 8. Effect of modulating DLK1 signaling in CMs, related to Figure 6.**

**A.** GSEA results showing representative enriched signaling pathways between 2D_CM_DLK1(2D_CM treated with sDLK1) and 2D_CM. **B.** Dotplots showing enriched activated signaling pathways in 2D_CM_DLK1 through GSEA. **C.** Dotplots showing enriched GO terms of upregulated genes (723 genes, Log_2_ fold change > 1) in 2D_CM_DLK1 vs. 2D_CM. **D.** Dot plots showing enriched GO terms of downregulated genes (771 genes, Log_2_ fold change > 1) in 2D_CM_DLK1 vs. 2D_CM.

**Supplementary Figure 9. *In vivo* transplantation procedure and evaluation, related to Figure 7.** **A.** Schematic diagram summarizing the protocol for MCO generation and application in the rat MI model. **B.** Experimental design for MI transplantation. After experimentally induced MI, SD rats were administered PBS (Control group), 1×10^7^ hiPSC-CMs (2D_CM group), MCO derived from 1×10^7^ hiPSC-CMs (MCO_CM group), and MCO derived from 2.5 ×10^6^ hiPSC-ECs, 2.5 ×10^6^ hiPSC-SMCs and 5 ×10^6^ hiPSC-CMs (MCO_Mix). Sham group just underwent thoracotomy and cardiac exposure without coronary ligation. Echocardiographic studies were performed to evaluate cardiac performance 1 day and 28 days after transplantation. On day 28, all rats were euthanized and the hearts were prepared for histological analysis. **C.** Table showing statistical data of echocardiographic parameters (LVFS and LVEF) on day 28 post-MI.

**Supplementary Figure 10. Quality control for scRNA-seq data.** Number of genes, number of unique molecular identifiers (UMIs), and mitochondrial gene percentage is plotted for 2D_CM (A), EC (B), SMC (C), MCO_CM (D), and MCO_Mix (E) samples, respectively.
